# Tebrophen — An Old Polyphenol Drug with Anticancer Potential ^†^

**DOI:** 10.3390/molecules17077864

**Published:** 2012-06-28

**Authors:** Ivica Rubelj, Višnja Stepanić, Dubravko Jelić, Nikolina Škrobot Vidaček, Andrea Ćukušić Kalajžić, Milena Ivanković, Krunoslav Nujić, Mario Matijašić, Donatella Verbanac

**Affiliations:** 1 Laboratory for Molecular and Cellular Biology, Division of Molecular Biology, Ruđer Bošković Institute, Bijenička cesta 54, HR-10000 Zagreb, Croatia; 2 GlaxoSmithKline Research Centre Zagreb Ltd., Prilaz baruna Filipovića 29, HR-10000 Zagreb, Croatia; 3 Laboratory for Epigenomics, Division of Molecular Medicine, Ruđer Bošković Institute, Bijenička cesta 54, HR-10000 Zagreb, Croatia; 4 Galapagos istraživački centar d.o.o., Prilaz baruna Filipovića 29, HR-10000 Zagreb, Croatia; 5 University of Zagreb, School of Medicine, Center for Translational and Clinical Research, Šalata 2, HR-10000 Zagreb, Croatia

**Keywords:** tebrophen, anticancer, high-throughput screening, molecular modelling, population doublings

## Abstract

*In vitro* high-throughput screening was carried out in order to detect new activities for old drugs and to select compounds for the drug development process comprising new indications. Tebrophen, a known antiviral drug, was found to inhibit activities on inflammation and cancer related targets. In primary screening this semisynthetic halogenated polyphenol was identified to inhibit the activities of kinases ZAP-70 and Lck (IC_50_ 0.34 µM and 16 µM, respectively), as well as hydrolase DPPIV (at 80 µM 41% inhibition). Next, it showed no cytotoxic effects on standard cell lines within 24 h. However, tebrophen slowed propagation of breast cancer (MDA-MB-231), osteosarcoma (U2OS) and cervical carcinoma (HeLa), through at least 35 population doublings in a dose-dependent manner. It completely stopped the division of the prostate cancer (PC3) cell line at 50 µM concentration and the cells entered massive cell death in less than 20 days. On the other hand, tebrophen did not influence the growth of normal fibroblasts. According to the measured oxidative burst and estimated *in silico* parameters its direct antioxidative ability is limited. The obtained results indicate that tebrophen can be considered a promising lead molecule for generating more soluble derivatives with specific anticancer efficacy.

## Abbreviations

BDE: bond dissociation enthalpy
HAT: hydrogen atom transfer
HTS: high-throughput screening
IP: ionization potential
PDs: population doublings
PMA: phorbol-12-myristate acetate
RSA: radical scavenging activity
SET: single electron transfer
TEB: tebrophen

## 1. Introduction

**Figure 1:**
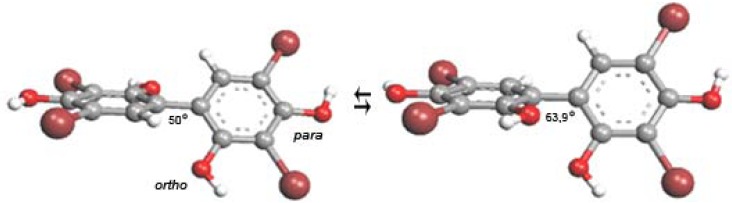
Polyphenolic structure of tebrophen (3,3',5,5'-tetrabromobiphenyl-2,2',4,4'-tetrol).The *trans* (left) and *cis* conformers are of similar stability.

In order to acquire new drug applications, systematic screening of a unique compound library [1], was carried out on different biological targets [2]. This high-throughput screening (HTS) started *in silico* and proceeded *in vitro* [3,4]. By applying this approach, tebrophen (3,3',5,5'-tetrabromobiphenyl-2,2',4,4'-tetrol, Figure 1), a drug known for the treatment of viral eye diseases, was found to inhibit activities of inflammation and cancer related targets, such as tyrosine kinases Lck and ZAP-70, and hydrolase Dipeptidyl peptidase IV (DPPIV/CD26), recently extensively studied [5].

Non-receptor tyrosine kinases are important components of signalling pathways [6]. Lck, p56 kinase participates in T-cell signal transduction [7,8,9]. Immunoreceptor Tyrosine Activation Motif (ITAM)-sequences of CD3 subunit and ζ-chain of T-cell receptor are phosphorylated by Lck. This phosphorylation is a prerequisite for ZAP-70 kinase activity, binding to ITAM and subsequent phosphorylation of proteins in the cascade that enables further downstream signalling. The final result of this process is proliferation and differentiation of T-cells, particularly due to overexpression of interleukine-2 (IL-2) and other cytokines [10]. Knowledge about ZAP-70 expression in malignant neoplasms [11] is still scarce. ZAP-70 is expressed by many lymphoma types and correlates with the immunoglobulin heavy-chain variable region gene mutational status in chronic leukemia and in non-Hodgkin and Hodgkin lymphoma. Polyphenols have been reported to have multiple effects on these and related kinases (Src and cyclin-dependent) and represent valuable starting point for further investigations of potential therapeutic interventions in this sense [12]. Our experience in HTS assessment of the inhibitory effects of polyphenolic compounds on kinases [13,14], was valuable for the verification and follow-up of the tebrophen afterward screening.

The peptidase DPPIV is also important in T cell activation, immune functions, signal transduction and apoptosis [15,16]. It interacts with antigen presenting cell, and regulates cytokine and chemokine function [17]. DPPIV plays an important role in diabetes, obesity, anxiety, rheumatoid arthritis, multiple sclerosis, cancer, autoimmune diseases and AIDS [5,18,19,20]. It acts by removing N-terminal dipeptides from proline-containing peptides such as incretins [21,22], some neuropeptides and vasoactive peptides [23,24]. This enzyme was extensively screened and investigated by the authors, due to its pleiotropic roles [3]. Recently, polyphenols have been reported to inhibit DPPIV at the μM level [25].

The drug tebrophen (Figure 1) was a very promising hit after all screening and validation procedures. Moreover, the compound was not cytotoxic in the standard cell assays after 24 h, but it has shown very interesting activity in cell based and functional assays applied to the determination of potential antiproliferative/anticancer properties. In order to assess cell specificity, we investigated the influence of tebrophen on the growth dynamics [population doublings (PDs)] of normal human fibroblasts and of a few specific cancer cell lines in which inhibition of such targets may reduce or even stop their propagation. The observed antiproliferative activity of tebrophen, against prostate cancer cells particularly, is likely specific, target related according to *in silico* and *in vitro* estimations of its limited direct antioxidative capacity.

## 2. Results

### 2.1. Docking Pose of Tebrophen within DPPIV Catalytic Site

**Figure 2:**
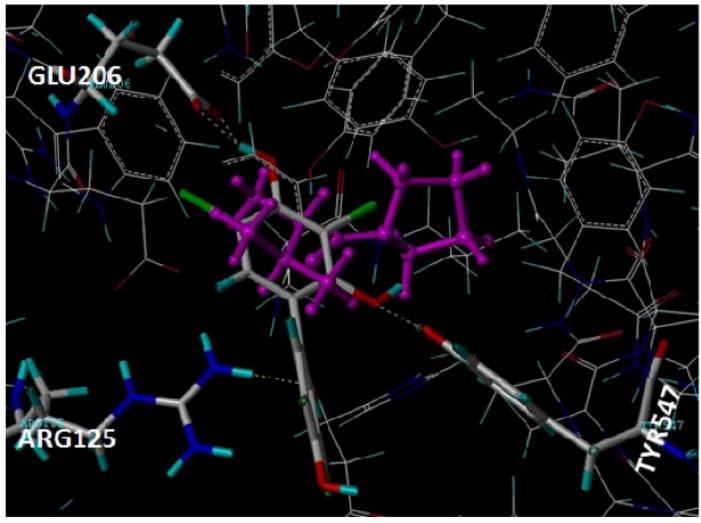
Molecular binding of tebrophen within the active site of DPPIV in comparison with the inhibitor valine-pyrrolidide coloured in magenta (PDB 1N1M [26]).

By the FlexX/DrugScore molecular docking procedure tebrophen was predicted to bind within the catalytic site of DPPIV placed at the interface of its β-propeller and α/β-hydrolase domains [26]. Although not occupied the S1 pocket close to the DPPIV catalytic Ser630-Asp708-His740 triad, tebrophen was recognized by H-bond interactions with DPPIV residues Glu205-Glu205 motif, Tyr547 and Arg125 of the S2 pocket (Figure 2). It was, however, scored lower as compared with the reference inhibitor valine-pyrrolidide as well as other competitive reversible non-covalent inhibitors [3].

### 2.2. *In Vitro* Inhibition of ZAP-70, Lck kinase and DPPIV

**Figure 3:**
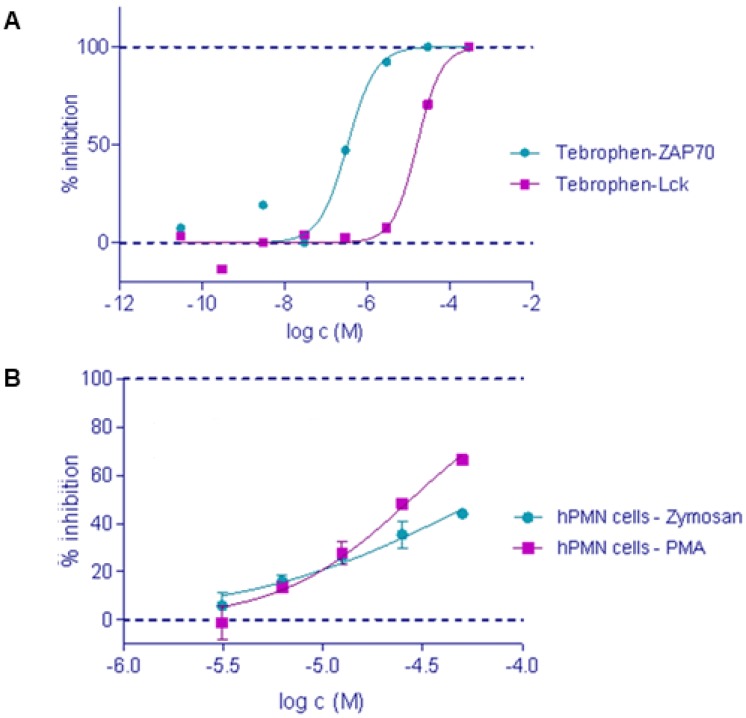
Tebrophen inhibits (**A**) ZAP-70 and Lck kinase activities with IC_50_ values of 0.34 μmol/L and 16 μmol/L, respectively and (**B**) oxidative burst stimulated with PMA or zymosan with IC_50_ values of 28 μmol/L and 62 μmol/L, respectively.

According to the results obtained using the standard ELISA method for kinases [27,28], tebrophen showed inhibition of ZAP-70 and Lck tyrosine kinase activities. It inhibited ZAP-70 and Lck kinase activities in a dose response manner at μM levels (Figure 3A). Tebrophen inhibition of Lck kinase was similar to the one obtained for natural flavonoids apigenin, myricetin, and quercetin [13]. Inhibition of DPPIV activity by tebrophen at 80 µM concentration was 41%.

### 2.3. Oxidative Burst

Influence of tebrophen on oxidative burst upon stimulation with PMA or zymosan [29] was measured *in vitro* on human polymorphonuclear (PMN) cells. The luminescence signal showed μM inhibitory effect of tebrophen on oxidative burst in a dose response manner (Figure 3B).

### 2.4. Cytotoxicity Assay

The *in vitro* cellular MTS cytotoxicity test [30] showed no significant inhibition of cell proliferation/metabolism within 24 h for the human (THP-1 and HepG2) and animal (CHO, COS-7) standard cancer cell lines, by Tebrophen in concentrations up to 50 μM (data not shown, available upon request).

### 2.5. The Effects of Tebrophenon on Cell Cultures Proliferation

#### 2.5.1. Normal Human Skin Fibroblasts NF (Primary Cells)

**Figure 4:**
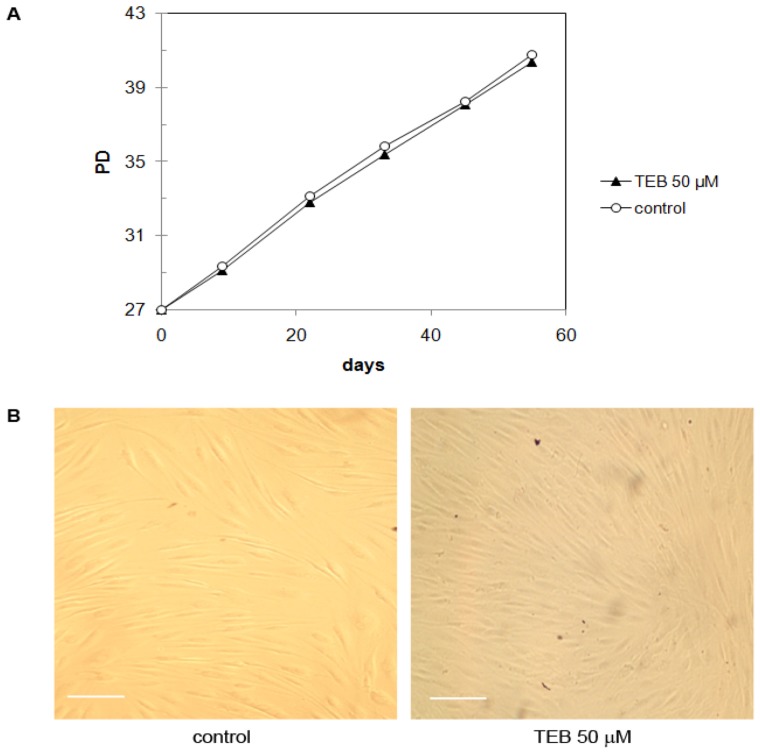
(**A**) Growth of NF fibroblasts starting at PD 27. Tebrophen (TEB) at 50 µM concentration did not influence NF fibroblasts growth rate as well as (**B**) their viability and morphology. Cells were documented at PD 34. Bar is equivalent to 100 µm.

Growth of the normal human skin fibroblasts NF [31,32] was followed in the presence of 50 µM tebrophen or 1% DMSO solvent only as a control. These cells reached senescence at PD ~55 (Figure 4A). For this experiment cells were seeded at PD 27 and maintained in the culture until PD 41. During this period they demonstrated a young phenotype and exponential growth. Tebrophen at 50 μM concentration did not influence NF fibroblasts growth rate or viability (Figure 4B). The NF fibroblasts also maintained normal morphology and showed no signs of stress over this period (~14 population doublings).

#### 2.5.2. Neonatal Foreskin Fibroblasts MJ90 (Primary Cells)

**Figure 5:**
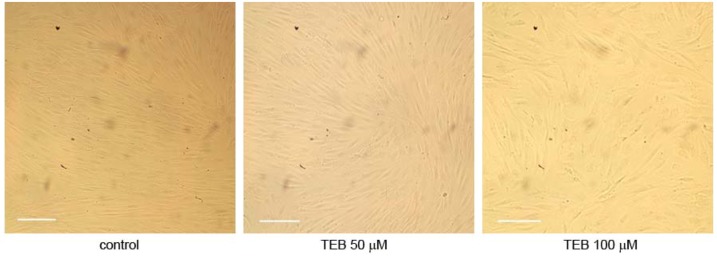
Growth of normal fibroblasts MJ90 in culture under standard conditions without tebrophen (TEB) and with 50 μM and 100 μM concentrations of tebrophen over 8 days. Bar is equivalent to 100 µm.

We also performed preliminary growth analysis of normal human male fibroblasts MJ90 over 8 days in culture. MJ90 neonatal foreskin fibroblasts were obtained from the Pereira-Smith laboratory (Barshop Institute for Longevity and Aging Studies, UTHSCSA, San Antonio, TX, USA) [31]. Cells were grown in culture under standard conditions over 8 days in the presence of 0 μM (control), 50 μM tebrophen and 100 μM tebrophen when photographed (Figure 5). During this time control cells and cells under 50 μM tebrophen reached confluency, showed no difference in growth potential (2.35 and 2.23 PD respectively) or morphology. Cells under 100 μM tebrophen showed reduced growth potential (1.46 over prolonged growth of 11 days) and moderately enlarged morphology.

Following observation that tebrophen has no effect on proliferation and viability of normal human skin fibroblasts in physiologically tolerant concentration (50 μM), we focused our interest on tebrophen as a potential anticancer agent. We studied the effect of tebrophen on a few frequently used cancer cell lines such as MDA-MB-231 [33,34], PC3 [35], U2OS [32,36] and HeLa [37,38].

#### 2.5.3. Breast Adenocarcinoma MDA-MB-231 Cell Line

**Figure 6:**
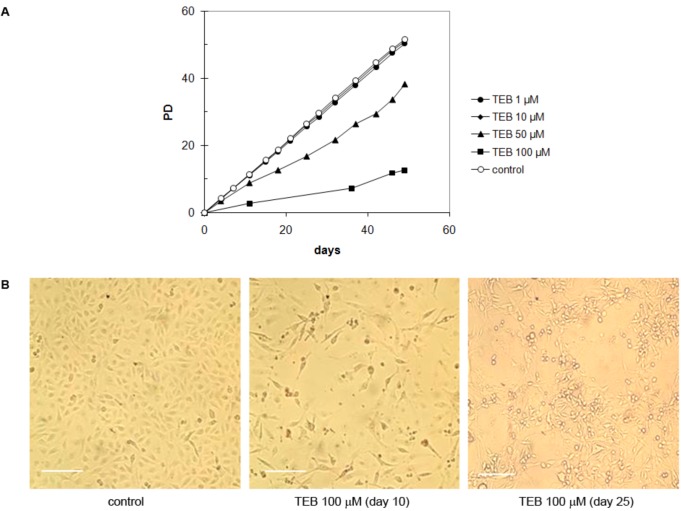
(**A**) Growth of MDA-MB-231 cells in the presence of tebrophen (TEB) at various concentrations. Growth is expressed in terms of population doublings (PD) with respect to the starting concentration at day 0. (**B**) Control with 1% DMSO only (left). Dead cells observed by microscope emerging at day 10 in the culture at 100 μM tebrophen (center). New focuses of growth of MDA-MB-231 cells in the presence of 100 µM tebrophen started to emerge after ~25 days in culture without reseeding (right). Bar is equivalent to 200 µm.

MDA-MB-231 cell line is derived from breast adenocarcinoma and has epithelial morphology [33]. At low concentrations (1 and 10 μM) tebrophen did not influence growth of MDA-MB-231 cells (Figure 6A). At 50 μM concentration, inhibition was significant, although cells continued to propagate at a slow rate. In the presence of 100 μM tebrophen, cells virtually seized divisions over long periods of time. At this concentration, at the beginning of treatment they significantly reduced in number [Figure 6A,B (center)]. However, they slowly recovered so that sporadic foci of growth started to emerge in the culture after ~25 days [Figure 6B (right)].

**Figure 7:**
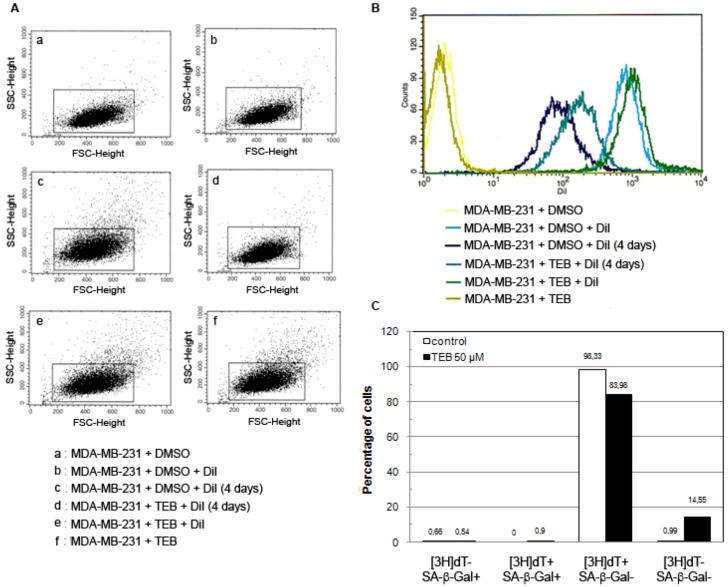
DiI FACS analysis of MDA-MB-231 cell growth with or without 50 µM tebrophen (TEB). (**A**) Gating strategy and sorted cells analysis. Bounded regions were drawn around cells according to their morphological properties on a FSC/SCC dot plot. Live cells were gated while dead cells and cell debris were excluded from further analysis and sorting. (**B**) DiI fluorescence intensity of positive control cells stained on the day of the experiment (light blue and green) and unstained negative control cells (yellow and olive). Control cell (dark blue) and cell treated with 50 μM tebrophen (turquoise) propagation after 4 days in culture. (**C**) Tritiated thymidine labeling index and SA-β-Gal activity in MDA-MB-231 control cells (red) and cells treated with 50 μM Tebrophen (blue). Four different phenotypes are described: [3H]dT+/SA-b-Gal−; [3H]dT−/SA-b-Gal+; [3H]dT+/SA-b-Gal+; [3H]dT−/SA-β-Gal−. At least 1,000 cells per sample were counted for statistical analysis.

The observed reduced growth rate could be a result of growth inhibition of one fraction of cells due to, for example, various levels of telomerase expression as it has been described in HeLa [37,39] or tebrophen could equally inhibit growth of all cells in the culture. In order to address this question, we investigated dynamics of MDA-MB-231 cells growth with or without tebrophen using DiI staining FACS analysis as well as SA-β-galactosidase staining and 3H-thymidine labeling index [31,40] (Figure 7). FACS analysis demonstrated that the entire population of MDA-MB-231 cells showed reduced growth rate (Figure 7A,B). Radioactive labeling index and SA-β-Gal confirmed inhibited growth rate since integration of radioactivity was reduced from 98.33% in control cells to 83.98% in tebrophen treated cells during 24 h (Figure 7C). 

At the same time tebrophen treated cells showed 14.55% of [3H]dT-/SA-β-Gal-phenotype due to slowed or postponed cell cycle. SA-β-Gal activity could be induced in immortal cell lines as a result of oxidative stress or DNA-damaging agents [41,42]. Since SA-β-Gal staining was very low in both cell samples, the observed effects of tebrophen could not be assigned to induced oxidative stress in these cells.

#### 2.5.4. Strong Inhibitory Effect of Tebrophen on Proliferation of Prostate PC3 Cell Line

**Figure 8:**
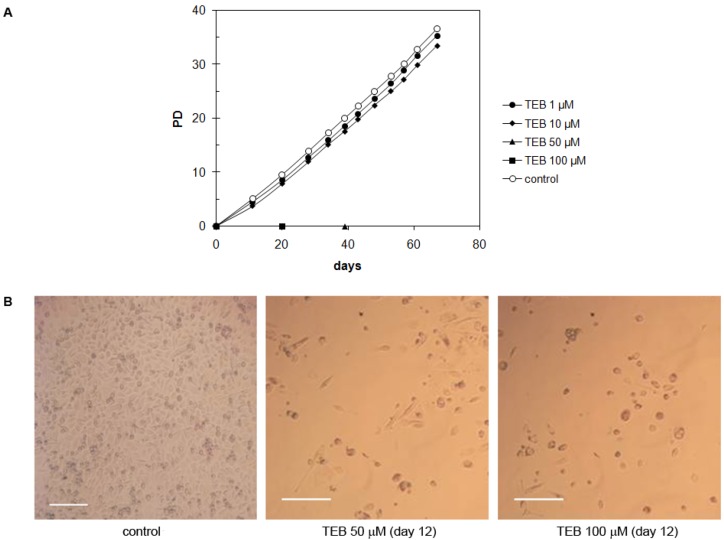
(**A**) Growth of PC3 cells in the presence of tebrophen (TEB) at various concentrations. Cells at concentrations of 50 and 100 µM of tebrophen died out and were cleared from the culture within 39 and 19 days, respectively. (**B**) Dead cells observed by microscope at day 12 in the culture at 50 and 100 µM tebrophen. Bar is equivalent to 200 µm.

The PC3 cell line is derived from prostate adenocarcinoma and has epithelial morphology [35]. These cells did not demonstrate significant inhibition of the growth rate at low concentrations of tebrophen (1 or 10 μM) over ~35 PDs (Figure 8A). On the contrary, at 50 or 100 µM tebrophen PC3 cells stopped dividing and massive cell death occurred. Their number continuously declined (Figure 8B) and they eventually completely vanished from the culture after 39 or 19 days, respectively.

Significant inhibition of the growth rate of MDA-MB-231 cells and complete clearance of PC3 cells from the culture was somehow unexpected since tebrophen had no significant effects on the viability of standard cell lines HepG-2, THP-1, CHO and COS-1 and the growth of NF fibroblasts up to ~55 days. We wanted to check whether this inhibitory effect of tebrophen was cell specific, so additional tests on well-known cell lines U2OS and HeLa were performed over a longer period of time (30–40 days in culture).

#### 2.5.5. Bone Osteosarcoma U2OS Cell Line

**Figure 9:**
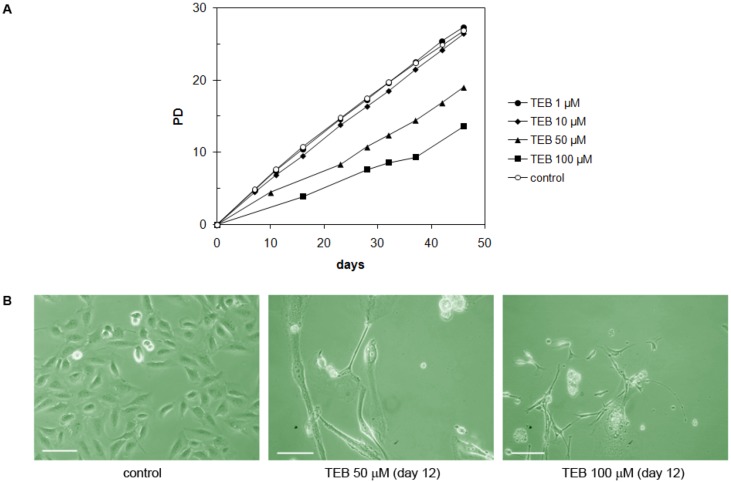
(**A**) Growth of U2OS cells in the presence of tebrophen (TEB) at various concentrations. (**B**) Control cells and cells treated with 50 and 100 µM tebrophen observed by microscope at day 12. Bar is equivalent to 100 µm.

The U2OS cell line is derived from bone osteosarcoma and shows epithelial morphology [36]. Tebrophen did not significantly influence growth rate of these cells at low concentrations (1 and 10 μM), but at 50 or 100 μM concentrations inhibition was significant (Figure 9A). These cells changed their morphology, enlarged and created extensions (Figure 9B). Nevertheless, since they retained constant growth although at low rate, we can say that overall these cells demonstrate substantial resistance to tebrophen.

#### 2.5.6. Cervical Adenocarcinoma HeLa Cell Line

**Figure 10:**
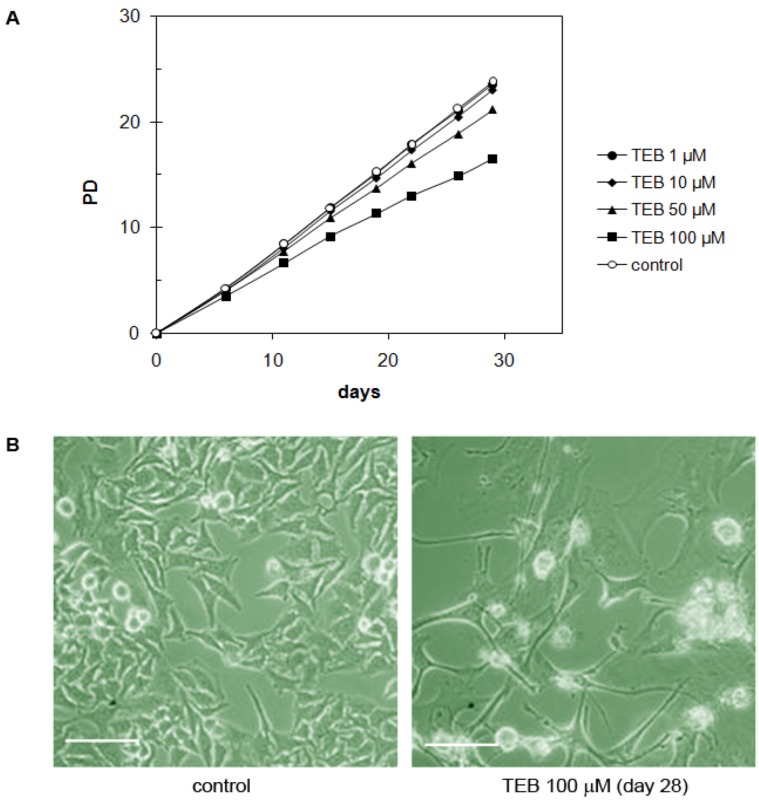
(**A**) Growth of HeLa cells in the presence of tebrophen (TEB) at various concentrations. (**B**) Control HeLa cells and cells with 100 μM tebrophen documented at day 28. Bar is equivalent to 100 µm.

The HeLa cell line is derived from cervical adenocarcinoma and has epithelial morphology [38]. They demonstrated very high resistance to tebrophen and grew well even in the presence of 50 or 100 µM tebrophen (Figure 10A). At 100 µM tebrophen, cells demonstrated some morphological changes such as enlargement and develop extensions. They kept enlarged phenotype throughout the experiment, but retained high viability and growth rate (Figure 10).

### 2.6. Radical Scavenging Activity of Tebrophen

The observed antiproliferative effect of tebrophen may be, at least partly, due to its antioxidative ability based on radical scavenging, similarly to other aromatic polyphenols whose radical scavenging activity (RSA) is due to their OH group(s). Non-enzymatic antioxidative ability of flavonoids, stilbenoids like resveratrol and plenty of other diverse polyphenolic compounds, is often related to their biological effects [43]. 

In order to test the hypothesis that RSA of tebrophen is responsible for its antiproliferative effect and due to the fact that there are no experimental data available for this molecule, the bond dissociation enthalpies (BDE) and acidities of the OH groups as well as molecular ionization potential (IP) were calculated and compared with the corresponding literature values [44] for the aromatic polyphenols showing antiproliferative effects on PC3 cancer cell line [45,46,47]. The calculated gas-phase BDE, IP and acidity values are collected in Table 1. In addition to plenty of developed *in vitro* assays [48] quantum-chemical calculations of these parameters have been extensively used for estimation of antioxidative capacity of chemical compounds [44]. The antioxidant properties of polyphenols may arise from direct reactions with various endogenous free radicals [44] and also from the chelation of free metals which changes metal redox potential and thus prevents it from taking part in the reactions generating free radicals [49]. Antioxidants can scavenge radicals in general, by the two major mechanisms:


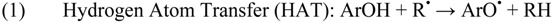


and:


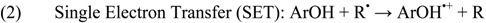


where R^•^ denotes a free radical. The HAT reaction is characterized by the O–H BDE parameter. A weaker O–H bond, that is a lower BDE, makes the phenolic hydrogen abstraction by a biological free radical more favourable. In the SET mechanism, the significant parameter for the scavenging activity evaluation has been assumed to be IP. The lower IP value of an antioxidant makes the electron release and radical reduction easier.

**Table 1:** Comparison of bond dissociation enthalpies (BDE), ionization potentials (IP) and acidities ^a^ of tebrophen and polyphenolic phytochemicals reported to induce apoptosis of PC3 cells. All calculated values are given in kcal/mol.

Species	BDE	IP	Acidity	ABTS^+^ assay (TEAC, mM) ^b^
*ortho*-Tebrophen	82.37	169.6	326.3	NA
*para*-Tebrophen	81.98	169.6	324.9	NA
Apigenin	82.2	176.0	321.3	0.086
Kaempferol	80.9	168.0	322.7	1.59
Luteolin	74.5	174.4		2.18
Quercetin	72.3	166.1	316.5	4.42
Resveratrol	77.3	161.3	327.5	2.14

^a^ The energetic parameters BDE, IP and acidities of tebrophen were calculated by using the same equations as in the reference [44]. For molecules apigenin to resveratrol the minimal O–H BDE and IP values calculated at the B3LYP/6-311++G(d,p) level are taken from reference [44]. The minimal O-H BDE/IP/Acidity values (in kcal/mol) calculated for e.g., quercetin, kaempferol and apigenin with the same model as for tebrophen are 74.2/166.2/319.7, 81.6/167.2/325.9 and 83.5/175.7/322.8, respectively. ^b^ Experimental activities are taken from reference [50].

Among listed bioantioxidants (Table 1), quercetin and resveratrol have the highest antioxidant ability as characterized by their lowest BDE and IP values [44,51]. Apigenin has poor radical quenching and reduction properties, while luteolin and kaempferol are of moderate antioxidant activities. In comparison, tebrophen is poorly susceptible to donate hydrogen atoms to free radicals. It may be more likely to scavenge free radicals by SET mechanisms since its IP is between IP values of good and weak antioxidants.

Since metal chelation by polyphenols often occurs through deprotonated hydroxyls, the compound acidity may play an important thermodynamic role [44]. The lower energy required to deprotonate the OH groups (less positive acidity parameter in Table 1), could result in more favourable metal chelating properties. Tebrophen is less acidic than most of the other polyphenols (Table 1). Although its acidity is similar to those of resveratrol which is known to form metal complexes [52], its reducing capacity was calculated to be weaker. According to the arrangement of hydroxyl and bromo groups (Figure 1), the metal chelating capacity of tebrophen can also be expected to be low.

Thus, tebrophen is estimated to be poorly active as just a hydrogen and electron donor. Non-enzymatic antioxidant capacity of tebrophen is quite unlikely to be responsible for its observed antiproliferative effects on PC3 cells. It should react with specific biological targets. As it has often been reported for polyphenols, tebrophen’s antioxidative activity may be due to its ability to modulate certain cellular signalling pathways like protein kinase pathways [43].

## 3. Discussion

The general scope of the drug discovery platform that finally led to the results presented here was to identify interactions between compounds from in house compounds library and pharmacologically relevant targets, using available *in silico* and biological *in vitro* screening approaches. The purpose was to recognize additional “hidden” values of in house compounds and to create conditions for the implementation of new program(s) into drug discovery pipeline, which is the approach widely accepted today by the pharmaceutical industry [53]. By combining computational and biological methods, positive and reliable hits were detected in a very short time frame by using small amounts of reagents. In this way the whole early stage drug discovery process known as the “hit generation” was performed utilizing minimal resources and any duplication work was avoided.

Further characterization of the best HTS compounds on cellular and functional assays indicated that an “old” drug, tebrophen (Figure 1) can exhibit quite significant interactions with “new” targets, and that the compound can be classified as a “promising hit” for the anti-inflammatory/cancer therapeutic area.

At first, we were encouraged with preliminary *in silico* screening result since tebrophen was successfully docked into DPPIV catalytic pocket (Figure 2), an enzyme of significant therapeutic interest [15,18,19,20,25]. This result was confirmed by *in vitro* detected inhibition. DPPIV/CD26 is a 110 kDa surface-bound ectopeptidase with intrinsic dipeptidyl peptidase IV (DPPIV) activity and multiple biological functions. As partially stated before, this enzyme modulates the biological functions of various peptides such as hormone peptides, neuropeptides and chemokines [23]. Apart from its catalytic activities, it interacts with proteins, for example adenosine deaminase (ADA), collagen, CD45 and the HIV derived protein -gp120. CD26 effects glucose metabolism through its DPPIV activity on glucagon-like peptide-1 (GLP-1). Its potential role as a therapeutic target in diabetes is of special significance from a clinical perspective [19,21,22]. It is expressed in many human tissues and is implicated in various biochemical processes, for example glucose homeostasis, activation of T lymphocytes, HIV infection and apoptosis, and it has a particularly important role in some chronic inflammation (e.g., rheumatoid arthritis) and neoplastic disease developments [5,18,54]. Some known antidiabetic drugs, such as metformin, have been considered as compounds with anticancer properties [55], acting through the kinase pathways, but when applying them in combination with cisplatin-based drugs, they can interfere with the cancer cells survival [56].

Accordingly, the class of targets included in our screening platform was tyrosine kinases. Protein kinases are a large family of enzymes that mediate the response of eukaryotic cells to external stimuli by phosphorylation of hydroxyamino acids. They play an essential role in many signaling pathways, and therefore have the potential to contribute to diseases ranging from cancer and inflammation to diabetes and cardiovascular disorders and many biological processes like cell growth and survival. Recent investigations have revealed that there are over 500 human kinases, making them the most populated class of targets, and many of them are targets for naturally derived compounds from plants, like polyphenols [12]. Phosphorylation of certain tyrosine residues changes the enzymatic activity of tyrosine kinases, thus regulating specificity for substrate binding, localization and recruitment of downstream signaling proteins. In primary single-target based screening, tebrophen was found to inhibit the two closely related kinases, Lck and ZAP-70, deriving from T-lymphocytes, in low μM range (Figure 3A). Results obtained for Lck were in the same range as previously reported results of testing natural polyphenols apigenin, myricetin and quercetin [13]. Assuming that structural basis for the inhibition of ZAP-70 activity might be similar to the that of cyclin-dependant kinases and Src kinases, which was later confirmed [57], and that many polyphenols like flavonoids have shown inhibition of Src and cyclin dependent kinases [47,58,59], logical consequence was further exploration of the potential of tebrophen, poorly investigated halogenated polyphenol considered as an antiviral drug [60]; in the framework of relevant therapeutic area. Tebrophen was considered as a non-cytotoxic compound according to the results obtained after its testing in standard cell lines (THP1-1, HepG-2, COS and CHO) applying MTS assays during 24 h.

In the oxidative burst assay, tebrophen exhibited an antioxidative effect, although not pronounced (Figure 3B). This result was confirmed by quantum-chemical calculations according to which tebrophen possesses no considerable radical scavenging and metal chelation activities (Table 1). Such results confirmed our observations that most probably tebrophen acts through interaction with specific target(s). 

Bearing in mind all results obtained, we decided to test the compound in functional and tailor-made cellular assays in order to access its capacity to stop proliferation of specific cancer cells in which DPPIV plays an important role acting particularly on glucagon-like peptide-1 (GLP-1) and glucose-dependent insulinotropic polypeptide (GIP), and to compare these results with other relevant cancer cell lines. We tried to apply a relevant approach to test the compound effect over a long period of time in parallel onto primary normal cell line and different cancer cell lines. This enabled us to build some conclusions on tebrophen specificity. Growth and viability of all considered cell lines were followed over extended periods of time (up to 50 PD) which could be relevant for potential application in therapy. In this respect, experiments on normal human skin fibroblasts NF or MJ90 (Figure 4 and Figure 5) did not show any difference in growth rate or sudden onset of cell senescence [61] over approximately 14 PDs in the presence of 50 µM tebrophen comparing to the control cells. This clearly demonstrates that the drug itself does not affect normal cell growth. 

On the contrary to normal cells, tebrophen interferes in cancer cell propagation. Inhibitory effects of tebrophen were investigated on growth and viability of the four cancer cell lines of various origins: MDA-MB-231 (breast cancer), PC3 (prostate cancer), U2OS (osteosarcoma) and HeLa (cervical carcinoma). These cells differed in their resistance to inhibitory effects of tebrophen. Most of them showed reduced growth rates, while PC3 underwent massive cell death and terminated culture propagation at tested concentrations of 50 and 100 µM tebrophen.

Low concentrations of tebrophen (1 or 10 µM) had no influence on the growth rate of MDA-MB-231 (Figure 6). At 50 µM concentration, the inhibitory effect of tebrophen was significant, but MDA-MB-231 cells remained viable and dividing. At 100 µM tebrophen, cells virtually ceased dividing over a long period of time (~25 days). During this period, most cells died out which dramatically reduced their number in the culture. However, some of them remained quiescent and eventually slowly recovered so that sporadic foci of growth started to emerge in the culture. Therefore MDA-MB-231 cells could undergo some modifications and regain ability to divide (Figure 7). We concluded that tebrophen has a moderate effect on the propagation of MDA-MB-231 cells.

In comparison to this, tebrophen did not show such strong effects on U2OS and HeLa cells. Precisely, low concentrations (1 and 10 µM) of tebrophen did not significantly influence growth of U2OS cells (Figure 9), although at 50 or 100 µM tebrophen inhibitory effects were more obvious. Overall U2OS cells demonstrated substantial resistance to tebrophen. HeLa cells also exhibited high resistance to tebrophen (Figure 10). They maintain high viability and growth rate even at the highest concentration of 100 µM tebrophen. 

The most interesting effect of tebrophen was observed on PC3 cells (Figure 8). They did not demonstrate very significant inhibition at low concentrations (1 or 10 µM), but at 50 or 100 µM of tebrophen present in the culture, they ceased division over long periods of time (about 39 or 19 days, respectively) and finally died. Thus, tebrophen has greater inhibitory effects on male cells (PC3) than on female cells (NF, HeLa, MDA-MB-231, U2OS) which could be attributed to hormone dependence of different cancer cells [62].

The results obtained with tebrophen on PC3 cells are in line with the results reported by Knowles *et al*. [47], where naturally occurring flavonoids have shown their complete retardation (using 100 μM quercetin) and suppression of growth (using apigenin and myricetin). In addition, not only natural (bromo)phenols, but also their synthetic analogs have also shown identical or even better *in vitro* protein tyrosine kinase inhibitory activity (μM) [63]. Thus, this could indicate pathway in searching for new chemical entities that could be further developed as therapeutic/chemopreventive agents for prostate cancer. 

## 4. Experimental

### 4.1. Tebrophen

Tebrophen was purchased from the InterBioScreen (Chernogolovka, Russia) and VitasM (Moscow, Russia) companies, and was prepared as 10^−2^ M stock solution using pure DMSO. 

### 4.2. In Silico

#### 4.2.1. Molecular Docking

The binding of tebrophen onto DPPIV was determined by a molecular docking procedure designed for *in silico* screening [3]. The calculations were carried out by the program FlexX, ver. 1.12.2 L [64] with its default setting. The docking was done by using the X-ray 2.5 Å structure of the extracellular region of DPPIV in complex with the inhibitor valine-pyrrolidide [26] (PDB code 1N1M [65]) and DrugScore scoring function [66]. The binding site was defined as a sphere with an origin at the hydroxyl O-atom of the catalytic amino-acid residue Ser630 and a radius of 10 Å. Input conformation of Tebrophen was pre-minimized by a Tripos force field with Gasteiger-Hückel charges and distance-dependent dielectric constant ε = ε(4r) (Tripos Inc., 2003) using a gradient of 0.05 kcal/mol Å as a terminating criterion.

#### 4.2.2. Quantum-Chemical Calculations

The equilibrium geometries of tebrophen in its unionized, oxidized radical and radical cation as well as anionic ground electronic states were fully optimized at the B3LYP/6-31+G(d,p) level in the gas phase. The minima on the potential energy surfaces were confirmed by vibrational frequency calculations. Both *ortho* and *para* hydroxyl (OH) groups were considered. All calculations were done with the Gaussian 03 program suite [67].

### 4.3. *In Vitro* ZAP-70 and Lck Kinase, and DPPIV Inhibition Protocols

ZAP-70 and Lck protein kinases were expressed in the Sf9 (*Spodoptera frugiperda* ovary cell) baculovirus expression system, and thereafter purified using chromatography methods. Purification and characterization were performed according to literature protocols [68,69,70].

The substrates *o*-phenylenediamine, HEPES, MgCl_2_, BSA, adenosine 5'-triphosphate disodium salt (ATP) and kinase substrate [random polypeptide copolymer Poly Glu:Tyr (4:1)], were obtained from Sigma (St. Louis, MO, USA). Peroxidase-labeled anti-phosphotyrosine antibody PY20 (mouse) was obtained from Calbiochem (San Diego, CA, USA). Tween 20 (polyoxyethylene sorbiton monolaureate) was obtained from Pharmacia (Uppsala, Sweden). MnCl_2_ and H_2_O_2_ were obtained from Merck (Whitehouse Station, NJ, USA). DTT (dithiothreitol) was obtained from Bio-Rad (Hercules, CA, USA). Measurements were performed by a Multiscan Ascent spectrophotometer (ThermoLabSystem, Helsinki, Finland).

*In vitro* inhibitions of ZAP-70 and Lck protein kinases by tebrophen were monitored according to the previously published ELISA method [4,13]. Briefly, phosphorylation of the substrate was monitored by an immunochemical reaction, where phosphorylated substrate residues were detected by specific immunocomplex and absorbance was measured at 490 nm. ELISA experiments were conducted in 96-well Dynex Immulon 2 HB microtiter plates (flat bottom, transparent). Peptide substrate in constant (10 µg/well) concentration was coated to the plate. After washing, buffer solution with ATP was added to each well. Compounds were added into wells in final doses that ranges from 10^−3^ mol/L to 10^−11^ mol/L. ZAP-70 and Lck protein kinases was added in 100 ng/well concentration and kinase reaction lasted 1 minute. After washing, blocking of the wells with 1% BSA in TBS was performed. Peroxidase-labeled anti-phospho-tyrosine antibody was then added and incubated for 40 min. After washing, peroxidase substrate [71] was added and the reaction was stopped after 20 min with 5 M H_2_SO_4_. Absorbance was measured at 490 nm. The IC_50_ value was calculated by using GraphPad Prism software, v. 3.02. 

DPPIV enzyme was obtained from R&D systems (Minneapolis, MN, USA), and activity of the compound was tested using H-Gly-Pro-pNA tosylate (Bachem, Bubendorf, Switzerland) as a substrate. The enzymatic reaction was performed in 100 μL of buffer containing 50 mM Tris, 150 mM NaCl at pH 7.9. The percent of inhibition was determined after 60 min.

### 4.4. Cytotoxicity Assay

Cytotoxicity assays on four different cell lines (THP-1, COS, HepG2, CHO) were run routinely as a part of secondary screening and compound profiling. Cancer cell lines were purchased from the ECACC: THP-1 (human monocyte leukemia) ECACC-88081201, HepG2 (human hepatocytes, epithelial) ECACC-85011430, CHO (chinese hamster ovary, epithelial) ECACC-85050302, and COS-7 (green african monkey kidney, fibroblasts) ECACC-87021302. Cells were maintained in complete RPMI 1640 medium (Institute of Immunology, Zagreb, Croatia) supplemented with 10% fetal bovine serum (BioWest, S04382S1810) at 37 °C in a 5% CO_2_ atmosphere. Cytotoxicity assay was performed by using the MTS CellTiter 96® AQueous One Solution Cell Proliferation Assay (G358B, 18824201, Promega, Madison, WI, USA). Each culture in 96-well plate contained 50 000 (for HepG2, CHO and COS-7 or 75000 for THP-1) cells. Cultures exposed to tested compounds were incubated for 24 h at 37 °C in 5% CO_2_. Thereafter, 15 μL of MTS reagent [30] was added directly to the cell lines. After an additional 2 h of incubation at 37 °C in 5% CO_2_, the absorbance was recorded at 490 nm using a spectrophotometric plate reader (Ultra, TECAN, Mannedorf, Switzerland). The method was programmed for the TECAN robotic system in GEMINI pipetting software [4].

### 4.5. Oxidative Burst Assay

The effect of tebrophen on oxidative burst was investigated *in vitro* with oxidative burst assay in human polimorphonuclear (PMN) cells. Assay was done in 96 well plates (Wallac, Turku, Finland, type “black & white”, flat bottom, 1450-581, 50,000 cells/100 μL of DMEM). The test compound was added to the cells in the final concentration of 50, 25, 12.5, 6.25 and 3.125 μM and mixed with 50 μL of luminol (5-amino-2,3-dihydro-1,4-ftalazindione, 0.25 mg/mL, Sigma, A-4685). Luminol as a detector of oxidative burst reacts with superoxide and light is produced as one of the reaction products. Subsequently, 50 μL of stimuli, phorbol-12-myristate acetate, PMA, 33 ng/mL, Sigma, P-8139, 125K1091) or zymozan (2 ng/mL, Sigma, Z-4250), were added. Signal was measured after 60 min of incubation of the cells with the test substances and stimulus. All experiments were done in duplicate. The control contained the same DMSO concentration as the test wells.

### 4.6. Functional Assays

#### 4.6.1. Cell Culture

Normal human skin fibroblasts NF [31], normal human skin fibroblasts MJ90 [32], HeLa cell line derived from cervical adenocarcinoma [38], U2OS cell line derived from bone osteosarcoma [36], MDA-MB-231 cell line derived from breast adenocarcinoma [33] and PC3 cell line derived from prostate adenocarcinoma [35] were grown in high glucose Dulbeco’s modified Eagle’s medium (DMEM; Sigma) supplemented with 10% fetal calf serum (Gibco, Grand Island, NY, USA) at 37 °C and 5% CO_2_. Following reseeding, cells were treated with 0, 1, 10, 50 and 100 μM of tebrophen and pure solvent dimethyl sulfoxide (DMSO, Sigma) as a control. When cells reached confluency they were trypsinized, counted for population doubling calculation and split for reseeding, usually in a 1:8 ratio. Population doubling (PD) was calculated according to the formula: PD = log (N_0_ cells harvested/N_0_ cells seeded)/log2. Any changes in cell morphology or growth were photographed for documentation.

Peripheral blood leukocytes were obtained from healthy volunteers (Department of Transfusion Medicine, Zagreb, Croatia). Fresh blood was mixed with dextran (5 mL 3% dextran T-500, Amersham Biosciences, Uppsala, Sweden, SAD/7 mL of blood), albumin (0.05% albumin A-6918, Sigma) and PBS with glucose (1.5 mL PBS with 0.18% glucose/7 mL of blood). After 30 min of sedimentation in 50 mL syringes, plasma was extracted. 

Neutrophils (PMN cells), were isolated by using fycoll (30 mL of plasma/15 mL fycoll; Ficoll-PaqueTMPlus, Amersham Biosciences, Uppsala, Sweden, 17-1440-02), after 30 min of spin on 1,800 rpm at room temperature. After removal of supernatant, PMN cells with erythrocytes were resuspended in 7 mL Milli-Q water, and 7 mL of 1.8% NaCl is added. After 10 min spin on 1,800 rpm at room temperature, supernatant was removed and PMN cells were re-suspended in 5 mL supplemented RPMI 1640 (Institute of Immunology, Zagreb, Croatia).

Peripheral blood mononuclear cells were isolated by using Histopaque 1077 (Sigma Diagnostics, St. Louis, MO, USA) or fycoll (15 mL Histopaque 1077/30 mL blood). After 30 min spin at 400 g, plasma above cells is removed. Peripheral blood mononuclear cells were collected with pipette, and diluted with PBS in clean tube. After 10 min spin on 1,300 rpm at room temperature, supernatant was removed and cells were resuspended in supplemented RPMI 1640 medium and fetal bovine serum (FBS). After determination of concentration, 500 μL of ice cold 20% DMSO in supplemented RPMI 1640 was added to the cells (1:1 ratio), and cells were frozen on −80 °C in concentration of 10 × 10^6^ cells/vial for future use.

#### 4.6.2. SA-β-galactosidase Staining and 3H-Thymidine Labeling Index

About 10^5^ cells were seeded in 25 cm^3^ bottle and incubated at 37 °C, 5% CO_2_. After approximately 24 h, 3H-thymidine was added at a concentration of 10 µCi/mL and incubation continued for another 24 h. Following this treatment, cells were fixed in 1% glutaraldehyde for 10 min, washed in PBS 2 × 5 min and stained for SA-β-Gal activity at pH 6.0 over 18 h as described previously [40,72]. For autoradiography, cells were washed 2 × 5 min in PBS, 2 × 5 min in 70% ethanol and dried at room temperature for several hours. In a dark room, fixed cells were overlaid with liquid photographic emulsion (Ilford Scientific Product, Knutsford, UK), wrapped in aluminum foil and stored at 4 °C for 48 h. Preparations were processed in standard Kodak developer and universal fixer. More than 1,100 cells were scored for statistical analysis.

#### 4.6.3. DiI Staining and Flow Cytometry

For fluorescent staining, an appropriate number of cells (1.5–2 × 10^6^) were trypsinized and spun down at 1,000 rpm in a swing-rotor laboratory centrifuge. Cells were than washed twice in PBS, resuspended in 10 mL of serum free DMEM medium containing 5 µM solution of DiI (1,1'-dioctadecyl-3,3,3',3'-tetramethylindocarbocyanineperchlorate, Molecular Probes, Eugene, OR, USA) and incubated for 20 min at 37 °C [31]. To remove excess of dye, cells were washed twice and cultured in the dark, wrapped in aluminum foil at 37 °C, 5% CO_2_. After 4 days in culture at ~80% confluence cells were trypsinized, washed twice in freshly prepared washing/staining buffer (PBS containing 2% FBS and 0.02% EDTA (Sigma) and resuspended in the same buffer at final concentration of 2 × 10^7^ mL^−1^. Cells were kept on ice until sorting. Positive control cells were stained and washed the same way on the day of sorting and placed on ice until sorting. Negative control cells were trypsinized and without staining resuspended in washing/staining buffer. Cells were analyzed on a Cell sorter (FASCCalibur, Becton Dickinson, Mountain View, CA, USA) using CellQuest Software (Beckton Dickinson). Two measured parameters, FSC (forward-scattered light) and SSC (side-scattered light) were detected with linear signal amplification. DiI specific fluorescence emission was detected on the FL2 channel using a 570/30 dichroic emission filter and logarithmic signal amplification. Results were analyzed by the Cell Quest Software program (Becton Dickinson).

## 5. Conclusions

By *in vitro* screening the already known antiviral drug, tebrophen has been revealed to inhibit tyrosine kinases-Lck and ZAP-70, and hydrolase-dipeptidyl peptidase IV at μM levels. This halogenated polyphenol has also been shown to be non-cytotoxic and to have no significant effects on proliferation of healthy primary human cells. However, it has been found to inhibit propagation of various immortal cells MDA-MB-23, PC3, U2OS and HeLa after longer period of time. Its different effects on various cancer cell lines could be attributed to specificity of these cells as they developed different surviving mechanisms of immortality [62] and since according to measurements and calculations it has no notable non-enzymatic antioxidant activity. Subsequently, while tebrophen eliminates PC3 cells and inhibits other studied cell line growth, it is suggested as a starting lead molecule for the design of more soluble derivatives with specific anticancer activity. For that purpose testing on several different prostate cell lines and detailed mechanistic study including tyrosine kinases [63] have to be performed. 
